# Comparison of adverse events between intensity-modulated radiation therapy and tomotherapy for early stage breast cancer: a retrospective cohort study

**DOI:** 10.3389/fonc.2025.1654609

**Published:** 2025-10-21

**Authors:** Yan Xia, Yan-Cheng Yang, Hang-Qi Ren, Yan-Zun Wang, Qing-Feng Li, Ya-Yuan Yu, Guang-Ran Yang, Yang-ke Li, Kai-Cheng Jin, Qi-Fa Luo, Zhi-Heng Bian, Tian Zeng, Jun-Qing Li

**Affiliations:** ^1^ Department of Oncology, Southwest Hospital, Army Medical University, Chongqing, China; ^2^ Department of Clinical Laboratory, The 990 Hospital of the Joint Logistics Support Force of the Chinese People’s Liberation Army, Zhumadian, China; ^3^ Department of Intensive Care Unit (ICU), The 942 Hospital of the Joint Logistics Support Force of the Chinese People’s Liberation Army, Yinchuan, China; ^4^ Department of General Surgery, Xin Qiao Hospital, The Second Affiliated Hospital, Army Medical University, Chongqing, China

**Keywords:** breast cancer, toxicity, adverse event, intensity-modulated radiation therapy, tomotherapy

## Abstract

**Introduction:**

Early stage breast cancer treated with adjuvant radiotherapy with two different techniques, tomotherapy (TOMO) and intensity-modulated radiation therapy (IMRT), and their acute adverse events in terms of skin toxicity, localized edema, sore throat, tracheal mucositis, nausea, oral mucositis, esophagitis, and pneumonitis outcomes are compared.

**Materials/methods:**

A retrospective cohort study was conducted to compare the adverse events of IMRT and TOMO in early stage breast cancer. We reviewed the data of female patients who underwent lumpectomy or mastectomy for breast cancer at the Oncology Department of the First Affiliated Hospital, Army Medical University, from September 2021 to February 2024. A total of 315 female patients were enrolled in this study, including 130 and 185 in the TOMO and IMRT groups, respectively. In this study, the adverse events in the two groups of patients were compared and analyzed.

**Results:**

The median age of the patients in this retrospective cohort was 47 years (range, 20–74 years). The follow-up period was 3 months. A total of 185 patients (59%) received IMRT and 130 (41%) underwent TOMO. No significant differences were observed in terms of menopausal status, laterality, pathology, estrogen receptor status, progesterone receptor status, triple negative, clinical T stage, clinical N stage, or surgical methods. Negative HER-2 overexpression was found in 38% and 51% of the TOMO and IMRT groups, respectively (relative risk [RR], 0.63; 95% CI 0.40 –0.99; P = 0.053).With regard to the degree of tumor differentiation, poor- moderate differentiation was 69% in the TOMO group and 81% in the IMRT group (RR 0.53; 95% CI, 0.31 –0.89; P = 0.052). In the TOMO and IMRT groups, 66% and 55% of the patients received hormone therapy, respectively (RR 1.59; 95% CI 1.00 –2.53; P = 0.5). However, there were no statistical differences in the demographic and tumor characteristics between the TOMO and IMRT groups. A comparison of adverse events between the TOMO and IMRT groups showed no significant differences in localized edema, sore throat, tracheal mucositis, nausea, oral mucositis, and the IMRT groups. Compared to the IMRT group, the TOMO group had a higher proportion of grade 3–4 skin toxicity [16.2% (TOMO) versus 7.6% (IMRT), (RR 2.13; 95% CI 1.04 –4.37; P = 0.017)]. Pneumonitis was lower in the TOMO group than in the IMRT group [0.0% (TOMO) versus 4.3% (IMRT), (RR 1.05; 95% CI 1.01 –1.08; P = 0.016].

**Conclusions:**

Compared with IMRT, TOMO decreases the incidence of radiation pneumonitis but fails to improve acute skin toxicity. Based on our research, TOMO may contribute to higher odds of acute skin toxicity, which should be considered by clinicians.

## Introduction

Breast cancer is a major disease that threatens women’s health (1). Breast cancer is the fourth leading cause of cancer mortality worldwide and the leading cause of cancer-related deaths among women (2, 3).

Radiotherapy has become one of the main methods used for adjuvant treatment of breast cancer (4, 5). Female patients with early stage breast cancer (stages 0 –II) commonly receive adjuvant radiation therapy after lumpectomy with or without (stage 0) sentinel node biopsy (6, 7). Adjuvant radiation showed a significant improvement in the local recurrence rate and overall survival rates in female patients with early stage breast cancer (8). However, female patients who received radiotherapy for breast cancer experienced different degrees of acute toxicity, such as tenderness or swelling of the chest wall, radiation dermatitis, radiation pneumonitis, and fatigue (9, 10).

In the past few years, toxic acute adverse events after radiotherapy for early stage breast cancer have been reported as an important issue (11, 12). According to the literature, tomotherapy (TOMO) has many advantages over precision radiotherapy, which contributes to the development and improvement of clinical treatment for breast cancer and minimizes the toxicity during radiotherapy. Precision radiotherapy strategies, including traditional intensity-modulated radiotherapy (IMRT) and TOMO, are the two main treatments for those female patients with early stage breast cancer after lumpectomy (13, 14).

Recently, many studies have revealed the toxicity of IMRT or TOMO radiotherapy for breast cancer (15, 16), but few studies have focused on the comparison of toxic side effects between IMRT and TOMO for early stage breast cancer. In particular, owing to the lack of direct comparative evidence on acute toxicity between TOMO and IMRT in early stage, node-negative breast cancer, it is challenging for clinicians to conduct a comprehensive comparison of the treatment effects and side effects between the two regimens. Therefore, physicians and patients urgently require more experiential support and evidence-based medical references for the selection of clinical treatment strategies for breast cancer radiotherapy. In this study, we aimed to evaluate the incidence of adverse events in the TOMO schedule compared with the IMRT schedule for early stage, node-negative breast cancer.

For this reason, we present our clinical experience using TOMO as an adjuvant radiation strategy for the early-stage breast cancer, and hope that would help improving the treatment strategies based on the results of this retrospective study. This study is also expected to provide a more optimized radiation strategy to decrease adverse events in female patients with early-stage breast cancer after lumpectomy or mastectomy.

## Materials and methods

A retrospective cohort study was conducted to compare the adverse events of TOMO and IMRT in early stage breast cancer. We reviewed the data of female patients who underwent lumpectomy or mastectomy for breast cancer at the Oncology Department of the First Affiliated Hospital, Army Medical University (Chongqing, China) from September 2021 to February 2024. A total of 315 female patients were enrolled in this study, including 130 in the TOMO group and 185 in the IMRT group. In this study, the adverse events of the two groups of patients were compared and analyzed. The study was approved by the internal ethics committee, and patient consent was obtained.

### Patient selection

In our clinic, patients who received adjuvant radiotherapy with TOMO or IMRT after surgery for early stage breast cancer in the Oncology Department of the First Affiliated Hospital, Army Medical University were evaluated retrospectively. Patient characteristics, treatment details, and acute adverse event data were obtained from the electronic medical records system, patient interview notes, and patient follow-up records. Acute adverse events in patients were evaluated by medical oncologists, radiation oncologists, and surgeons.

### Grouping methods

This study was designed as a single- center retrospective cohort analysis. All patients with early stage breast cancer who underwent lumpectomy or mastectomy between September 2021 and February 2024 were included in the primary analyses. According to the inclusion criteria, the patients were divided into two groups, the IMRT and TOMO groups, based on the radiotherapy regimens they received. After screening with the exclusion criteria, patients who met the above two criteria were included in the final analyses.

The eligibility criteria were as follows: age >18 years, invasive cancer, American Joint Committee on Cancer AJCC Stage I to II, lumpectomy or mastectomy, and TOMO or IMRT radiotherapy. The main exclusion criteria were extensive intraductal carcinoma, multiple foci of cancer, final surgical margins < 5 mm, lack of clinical data, vital organ failure, and failure to complete radiotherapy.

### Treatment planning

Radiation therapy was systematically prescribed following our institutional policy. Radiotherapy treatment was started 30 days and within 60 days from the surgery; if adjuvant chemotherapy was performed, radiotherapy was postponed until 4 weeks after the last chemotherapy cycle. Patients in different groups received radiation of the whole breast and/or surgical bed using two different devices, TOMO or IMRT. The treatment procedure followed institutional rules, which have been described in detail elsewhere (17, 18).

### TOMO planning

Treatment planning for TOMO was performed using the Accuray^®^ Planning Station System (TomoHDTM version 2.1.9, Inc., Sunnyvale, CA, USA). The Monte Carlo algorithm is used for dose calculation, treatment planning, and quality assurance. The grid size is 3 mm. In line with the internal irradiation regimes, the dose prescribed to the planning target volume (PTV) varied from 40 Gy to 60 Gy, with a median dose of 50 Gy for the PTV. Before radiation treatment, the patient’s positioning in each automatic registration was executed by experienced staff members (see **Supplementary File 1**, detailed protocols of treatment planning).

As a newly introduced therapy equipment, TOMO is more expensive than that of IMRT. TOMO was chosen for some patients mainly based on their economic conditions and fully complied with the patient’s voluntary choice.

### IMRT planning

We utilized the Eclipse version 16.1 (Varian Medical Systems Inc., Palo Alto, USA) to create IMRT treatment plans. Patients undergoing IMRT were administered a cumulative dose of 50 Gy in 25 fractions. Subsequently, a radiation therapy boost of 10 Gy was administered in five weekly fractions to the surgical bed. The dose was delivered through wedged photon tangential fields, and the boost was treated using an electron direct field. The organs at risk (OARs) were contoured according to internal guidelines. The constraints specified that 5% of the heart and 20% of the lung should receive a dose of less than 20 Gy (see **Supplementary File 1**, detailed protocols for treatment planning).

### Follow-up

The follow-up period was 3 months. After completion of TOMO or IMRT radiotherapy, according to the research plan, the follow-up schedule was as follows: we followed up all patients weekly for 3 months. The start date of follow-up for each patient was the radiation therapy start date, and the end date was 3 months after the last radiation therapy date. Follow-up mainly depended on the outpatient department, and telephone contact was reserved as an auxiliary method. Clinical examinations were performed by clinicians at each follow-up visit, and other examinations, such as hematologic or endoscopic examinations, were performed depending on the patients’ suspected symptoms. Adverse events were diagnosed by clinicians according to objective clinical and physical examinations after radiotherapy during treatment and follow-up. This study was completed in February 2024.

### Outcomes

The clinical endpoint of this study was acute adverse events immediately following the completion of radiation therapy. Patients’ acute adverse events were prospectively recorded for a period of 3 months and were evaluated by medical oncologists, radiation oncologists and clinicians in accordance with the Common Terminology Criteria for Adverse Events (CTCAE) (Version 5.0) (19). In this study, acute adverse events were defined as those first observed and diagnosed within 90 days of the latest radiotherapy session. We recorded acute skin toxicity (erythema, epilation, desquamation, decreased sweating, edema, ulceration, hemorrhage, and necrosis), localized edema, sore throat, tracheal mucositis, nausea, oral mucositis, esophagitis, and pneumonitis. Adverse events were recorded weekly during TOMO or IMRT treatment and then repeated until 3 months after the last radiotherapy. Particularly, before the implementation of treatment, clinicians assessed the skin condition to ensure that the skin was clear, normal, and with out lesions. Patients were also excluded from the study if they had any risk factors (such as comorbidities or concurrent medications) for vulnerable skin.

### Statistical methods

This study was designed to compare the toxicity rates of IMRT and TOMO in patients with early stage breast cancer after lumpectomy or mastectomy. Statistical analyses were performed using the SPSS Statistics software (version 26; SPSS Statistics, IBM Corporation, Armonk, NY, USA). Independent t-tests, chi-square tests, and Fisher’s exact tests were used to compare the statistical differences between the two groups. Econometric data that conformed to normal distribution with homogeneous variances were expressed as mean ± standard deviation () and subjected to t-tests. Count data were expressed as the number of cases (percentage) N (%), and intergroup comparisons were performed using the X^2^-test or Fisher’s exact test. All 2-sided P values <0.05 were considered significant.

## Results

**Figure 1:**
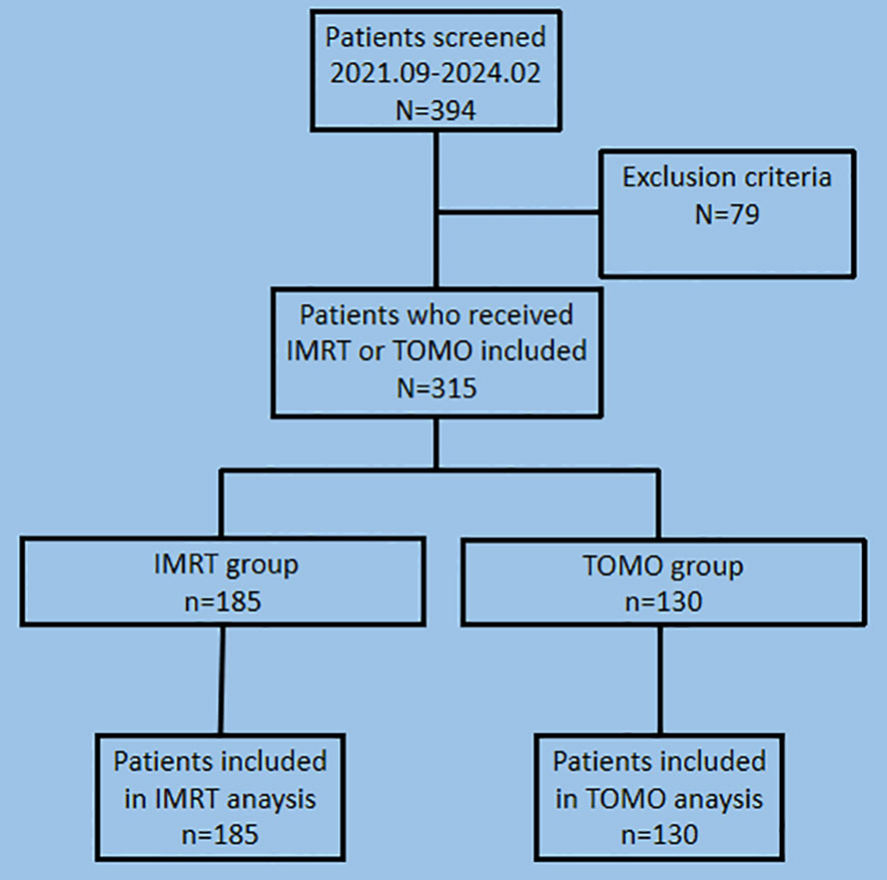
Screening and patient flow in the study of adverse events comparison between TOMO and IMRT for early stage breast cancer. TOMO, tomographic intensity-modulated radiation therapy; IMRT, intensity-modulated radiation therapy.

Between September 2021 and February 2024, 394 patients with early stage breast cancer who underwent lumpectomy or mastectomy were included in the primary analyses. After screening patients with the exclusion criteria, 79 patients were excluded, and 315 patients were enrolled in this study. The TOMO group and IMRT groups comprised 130 and 185 patients, respectively (**Figure 1**).

### Clinical characteristics

The median age of the patients in this retrospective cohort was 47 years (range, 20–74 years). **Table 2** summarizes the clinical characteristics of the 315 patients, divided by planning method into IMRT and TOMO. The length of follow-up was 3 months. 185 patients (59%) received IMRT and 130 patients (41%) underwent TOMO. Negative HER-2 overexpression was found in 38% and 51% of the TOMO and IMRT groups, respectively (RR 0.63; 95% CI 0.40 –0.99; P = 0.053).With regard to the degree of tumor differentiation, poor-moderate differentiation was 69% in the TOMO group and 81% in the IMRT group (RR 0.53; 95% CI 0.31 –0.89; P = 0.052). There were 66% and 55% of TOMO and IMRT groups, respectively, receiving hormone therapy (RR 1.59; 95% CI 1.00 –2.53; P = 0.5). However, there were no statistical differences in the demographic and tumor characteristics between the TOMO and IMRT groups.

The baseline clinical characteristics were well-balanced between the TOMO and IMRT groups (**Table 1**), and no significant differences were observed in terms of age, menopausal status, laterality, pathology, estrogen receptor status, progesterone receptor status, triple negative, clinical T stage, clinical N stage, or surgical methods.

**Table 1:** Description of acute adverse events related to radiotherapy.

Acute adverse events	IMRT	TOMO	p-Value
	(n = 185)	(n = 130)	
Skin toxicity			
0–2	171 (92.43%)	109 (83.85%)	0.017
3–4	14 (7.57%)	21 (16.15%)	
Localized edema			
0	4 (2.16%)	0 (0%)	0.092
1	181(97.84%)	130(100%)	
Sore throat			
0	176(95.14%)	124(95.38%)	0.918
1-2	9(4.86%)	6(4.62%)	
Tracheal mucositis			
0	184(99.46%)	128(98.46%)	0.369
1-2	1(0.54%)	2(1.54%)	
Pneumonitis			
0	177 (95.68%)	130 (100%)	0.016
1–2	8 (4.32%)	0 (0%)	
Nausea			
0	182 (98.38%)	130 (100%)	0.145
1–2	3 (1.62%)	0 (0%)	
Mucositis oral			
0	180 (97.30%)	129 (99.23%)	0.216
1–2	5 (2.70%)	1 (0.77%)	
Esophagitis			
0	184 (99.46%)	127 (97.69%)	0.168
1–2	1 (0.54%)	3 (2.31%)	

TOMO, tomographic intensity-modulated radiation therapy; IMRT, intensity-modulated radiation therapy.

CTCAE grading: grade 0 = none, grade 1–2 = mild/moderate, grade 3–4 = severe.

**Table 2:** Comparison of clinical characteristics of patients between the TOMO and IMRT groups.

General characteristics	IMRT	TOMO	p-Value
	(n = 185)	(n = 130)	
Age			
Mean (years)	47.48 ± 10.21	47.41 ± 10.20	0.45
Range	20–74	24–72	
Menopausal status			
Premenopausal	95 (51.35%)	74 (56.92%)	0.329
Postmenopausal	90 (48.65%)	56 (43.08%)	
Laterality			
Right	88 (47.57%)	61 (46.92%)	0.91
Left	97 (52.43%)	69 (53.08%)	
Pathology			
Ductal carcinoma	95 (51.35%)	63 (48.46%)	0.867
Lobular carcinoma	6 (3.24%)	4 (3.08%)	
Others (Intraductal)	84 (45.41%)	63 (48.46%)	
Receptor status			
Estrogen receptor status			
Negative	51 (27.57%)	34 (26.15%)	0.781
Positive	134 (72.43%)	96 (73.85%)	
Progesterone receptor status			
Negative	103 (55.68%)	65 (50%)	0.320
Positive	82 (44.32%)	64 (49.23%)	
Uncertain	0 (0%)	1 (0.77%)	
HER2 overexpression			
Negative	94 (50.81%)	50 (38.46%)	0.053
Positive	91 (49.19%)	79 (60.77%)	
Uncertain	0 (0%)	1 (0.77%)	
Triple negative			
Yes	23 (12.43%)	8 (6.15%)	0.066
No	162 (87.57%)	122 (93.85%)	
Differentiation			
Poor-Moderate	150 (81.08%)	90 (69.23%)	0.052
Well	9 (4.86%)	8 (6.15%)	
Uncertain	26 (14.06%)	32 (24.62%)	
Clinical stage			
Clinical T stage			
0–1	113 (61.08%)	77 (59.23%)	0.543
2–4	61 (32.97%)	41 (31.54%)	
Uncertain	11 (5.95%)	12 (9.23%)	
Clinical N stage			
0–1	159 (85.95%)	107 (82.31%)	0.672
2-3	23 (12.43%)	20 (15.38%)	
Uncertain	3 (1.62%)	3 (2.31%)	
Treatment details			
Hormone therapy			
Yes	102 (55.14%)	86 (66.15%)	0.5
No	83 (44.86%)	44 (33.85%)	
Surgery			
Breast conservative surgery	105 (56.76%)	82 (63.08%)	0.261
Modified radical mastectomy	80 (43.24%)	48 (36.92%)	

TOMO, tomographic intensity-modulated radiation therapy; IMRT, intensity-modulated radiation therapy.

### Acute adverse events evaluation

The clinical results of adverse responses and comparisons between TOMO and IMRT are summarized in Table 3. There was no significant relationship between observed localized edema (RR 1.02; 95% CI 1.00 –1.04; P = 0.092); sore throat (RR 0.95; 95% CI 0.33 –2.73; P = 0.918); tracheal mucositis (RR 2.88; 95% CI 0.26 –32.04; P = 0.369); nausea (RR 1.02; 95% CI 1.00 –1.04; P = 0.145); Oral mucositis (RR 0.28; 95% CI 0.03 –2.42; P = 0.216); esophagitis (RR 4.35; 95% CI 0.45 –42.26; P = 0.168). Compared to the IMRT group, the TOMO group had a higher proportion of grade 3–4 grade skin toxicity [16.2% (TOMO) versus 7.6% (IMRT), (RR 2.13; 95% CI 1.04 –4.37; P = 0.017)]. Pneumonitis was lower in the TOMO group than in the IMRT group [0.0% (TOMO) vs. 4.3% (IMRT), (RR 1.05; 95% CI 1.01 –1.08; P = 0.016]]. No fair or poor judgments were recorded in the 315 patients during the follow-up period. No other adverse events or toxicities were observed during the follow-up period. The clinical results are summarized in **Table 1**.

On comparing adverse events, we found a statistically significant difference between the TOMO and IMRT groups in the presence of acute skin toxicity (P = 0.017) and pneumonitis (P = 0.016). Compared with the IMRT group, the TOMO group seemed to have a lower incidence rate of pneumonitis. However, the incidence of acute skin toxicity was higher in the TOMO group was higher than that in the IMRT group, especially for grade 3–4 skin toxicity. The results suggest that TOMO could decrease the incidence rate of pneumonitis but increase the risk of acute skin toxicity.

## Discussion

In the present retrospective, single-center study of 315 patients treated for breast cancer with IMRT or TOMO, we found that adverse events occurred very commonly (observed in 98.7% of the patients), and a considerable number of patients in this study suffered at least one (mainly mild) toxicity adverse event. Our study showed a notable improvement in reducing the incidence of radiation pneumonitis in the TOMO group. For the IMRT group, 4.3% of all patients developed radiation-related pneumonitis, but it was not severe (with only eight events of grade 2 or lower), while the incidence in the TOMO group was 0%. Similarly, studies (20, 21) have revealed that TOMO could decrease unnecessary breast overdose in breast-conserving treatment of breast cancer; as a result, TOMO decreased adverse events in some critical organs, such as the lungs, by optimizing ipsilateral lung dosimetry (21, 22).

In addition, in a single -center retrospective study, Felix et al. (23) discovered that TOMO presented low rates of acute toxicity in critical organs. Pneumonitis was observed in 1.8% of patients who received treatment (23). During the follow -up period, none of the patients experienced toxicities higher than grade 3. A recent retrospective study that investigated the clinical outcomes and adverse events associated with adjuvant radiotherapy using TOMO after breast -conserving surgery disclosed that the adverse events were mild, and there was no occurrence of pneumonitis in the observed patients (24).

A similar result was also obtained in our study, and we also found no pneumonitis in patients after TOMO. As mentioned above, TOMO improved the critical organ risk, especially for the lungs, during radiotherapy by optimizing treatment planning.

However, that study showed no improvement in other acute adverse events related to radiotherapy, such as localized edema, sore throat, tracheal mucositis, nausea, oral mucositis, and esophagitis. Notably, acute skin toxicity appeared to be more severe in the TOMO group. Although there was no statistical difference between the two groups in the incidence of skin toxicity (grade 0–4), unfortunately, skin toxicity grade 3–4 was significantly increased in the TOMO group. In the TOMO and IMRT groups, 16.2% and 7% of all patients had acute skin toxicity grade 3–4, respectively. Simon et al. (25) explained that if the skin surface is set as a radiation therapy optimization target, tangential beam segments would concentrate on the skin surface as a result of inverse planning, which would increase acute skin toxicity. The flexibility of TOMO in delivering doses to the tumor bed makes it easier to accumulate high doses to superficial targets, such as the skin, resulting in significant acute skin toxicity (26, 27). Theoretically, if the “hot-spot” (>10% of prescribed dose) of TOMO delivers an overdose on the skin surface, an abnormally high incidence of acute skin toxicity follows (26).

Moreover, according to the results previously reported in the literature, factors such as the TOMO planning system (27–29), patient positioning (27, 30), breast size variation (31), treatment delivery time (27), and edema or breath variation (32) contribute to the incidence of skin toxicity. Clinically, it is difficult to diminish the impact of these risk factors. For example, a patient positioning shift of 5 mm during TOMO may induce an extra dose variation of 3% –9% (30). In addition, different systems of TOMO planning software may contribute 3%–13% of overdose to skin tissue (29).

Although these risk factors for acute skin toxicity are difficult to overcome, additional care must be taken to ensure patient safety and prevent skin toxicity. According to the literature reported above, when treating breast cancer patients with TOMO, clinicians should pay more attention to ensure that patients are in accurate positioning (27). Meanwhile, optimized measurements or dose recalculation techniques should be applied to the TOMO planning software to ensure adequate dosing for superficial organs, including the skin, during radiation therapy (27, 32). Furthermore, more robust new techniques, including artificial intelligence, should be applied using TOM to reduce skin dose and avoid toxicity (25, 33–35).

In the present study, 21 (16.15%) patients in the TOMO group had severe (grade 3–4) skin lesions, while the data in the IMRT group was 14 (7.57%). Thus, TOMO results in a higher incidence of skin toxicity. When examining the underlying reasons, we are more inclined to attribute the higher incidence of skin toxicity to the unique mechanism of action of TOMO. The reasons are as follows: First, all treatment plans were performed by operators from the same group; therefore, instrument operation-related risk factors, such as patient positioning or treatment planning, should be excluded. Second, in our study, the sample size was relatively large for a single-center study, which could effectively mitigate the impact of patient heterogeneity on clinical treatment responses; consequently, the results in the present study are relatively reliable. Finally, in terms of skin toxicity, our research results were consistent with previous literature reports, which presented an abnormally high incidence of acute skin toxicity in their studies.

Few studies have reported adverse events associated with TOMO vs. IMRT in early stage breast cancer. This study reports our initial experience with postoperative radiotherapy using TOMO in breast cancer. In our experience, although we were not able to optimize the radiation dose on the skin tissue and reduce the incidence of acute skin toxicity, TOMO could still decrease radiation pneumonitis in early stage breast cancer after surgery. Furthermore, other clinical results also showed that the acute adverse events related to radiotherapy in TOMO were not inferior to those in IMRT, which suggested that compared to IMRT, TOMO may achieve similar or superior target coverage and better critical organ sparing.

### Limitation

We are aware of the limitations of our study. The retrospective, single-center design of this analysis might affect the interpretation of the data, and the persuasiveness of our conclusions was weaker than that of multicenter prospective studies. Moreover, although we enrolled patients strictly in accordance with the inclusion and exclusion criteria, the absence of randomization in group assignment might have led to potential selection bias.

In the present study, we focused only on eight types of adverse events, with a relatively small number of clinical indicators observed during radiotherapy. Furthermore, the primary goal of this data release is to retain as much clinical data as possible for reference and discussion among physician peers. Based on the above considerations, we did not apply strict multiple test correction methods, such as the Bonferroni correction, to adjust the p-values. Instead, we only performed limited statistical methods, such as t-test and chi-square analysis, which might increase the risk of false positives in the statistical results.

Due to the lack of experience in our work, we failed to precisely match the occurrence of adverse events with the corresponding follow -up periods while implementing the research plan. We only summarized the outcomes after the three-month follow-up was completed, which hindered our understanding of how time influenced the development of adverse events during or after radiotherapy. In other words, we were unable to leverage weekly follow-up data using longitudinal models to evaluate toxicity trajectories over time.

It is worth mentioning that a 3-month follow-up might be too short to capture the late effects of radiation therapy. Because we only focused on short-term complications at the initial stage of the study, a relatively short follow-up period was designed accordingly. Consequently, only acute toxicity complications were reported in our study, while long-term prognosis, including late effects and survival analysis results, were lacking due to the short follow-up duration.

Lastly, the relatively simplistic and limited statistical methods, such as the T-test and Chi-square analysis, employed in the study may compromise the persuasiveness of the results and weaken the robustness of the conclusions.

Future perspectives call for continued efforts to conduct more extensive studies with longer follow -up periods. In the next phase of our study, we will extend the follow-up period to 3 years and shift our focus to late adverse events and survival analysis to gain a better understanding of survival and long -term toxicity outcomes and provide valuable clinical research data and experience for radiotherapy treatment after breast cancer surgery.

## Conclusion

Compared with IMRT, TOMO decreases the incidence of radiation pneumonitis but fails to improve acute skin toxicity. The present experience of applying TOMO in radiotherapy for early stage breast cancer suggests that, with the exception of pneumonitis, it may not be conducive to decreasing acute toxicity adverse events in early stage breast cancer after lumpectomy or mastectomy. Based on our research, TOMO may contribute to higher odds of acute skin toxicity, which should be considered by clinicians. Clinicians should consider the balance between the benefits and risks of TOMO . However, long-term follow-up is needed to perform in order to assess chronic toxicity and survival outcomes after TOMO in early stage breast cancer.

## Data Availability

The raw data supporting the conclusions of this article will be made available by the authors, without undue reservation.

## Supplementary material

Supplementary File 1 Detailed protocols of treatment plan about TOMO and IMRT.
