# Supplementary material for: Comparison of adverse events between intensity-modulated radiation therapy and tomotherapy for early stage breast cancer: a retrospective cohort study
Source: Front Oncol. 2025 Oct 21;15:1654609. doi: 10.3389/fonc.2025.1654609 (PMC12582959; doi:10.3389/fonc.2025.1654609)
Supplement: Supplementary File 1 — Detailed protocols of treatment plan about TOMO and IMRT. [file DataSheet1.pdf]

TOMO planning

Treatment planning was performed with the Accuray® Planing Station System (TomoHDTM version 2.1.9, Inc., Sunnyvale CA, USA). The primary objective of treatment planning in radiation therapy is to deliver the prescribed dose uniformly to the planned target volume (PTV) while maximizing the protection of adjacent healthy tissues and organs at risk (OARs).

Contouring

For contouring the clinical target volume (CTV), a consistent 3-dimensional margin of 1 cm was established circumferentially around the surgical clips to encompass the intended target region, including I, II, and III groups of axillary lymph nodes and internal mammary nodes. A 5 mm margin between CTV and PTV was used for both breast and lymph nodes, the CTV was limited to 3 mm from the skin surface.

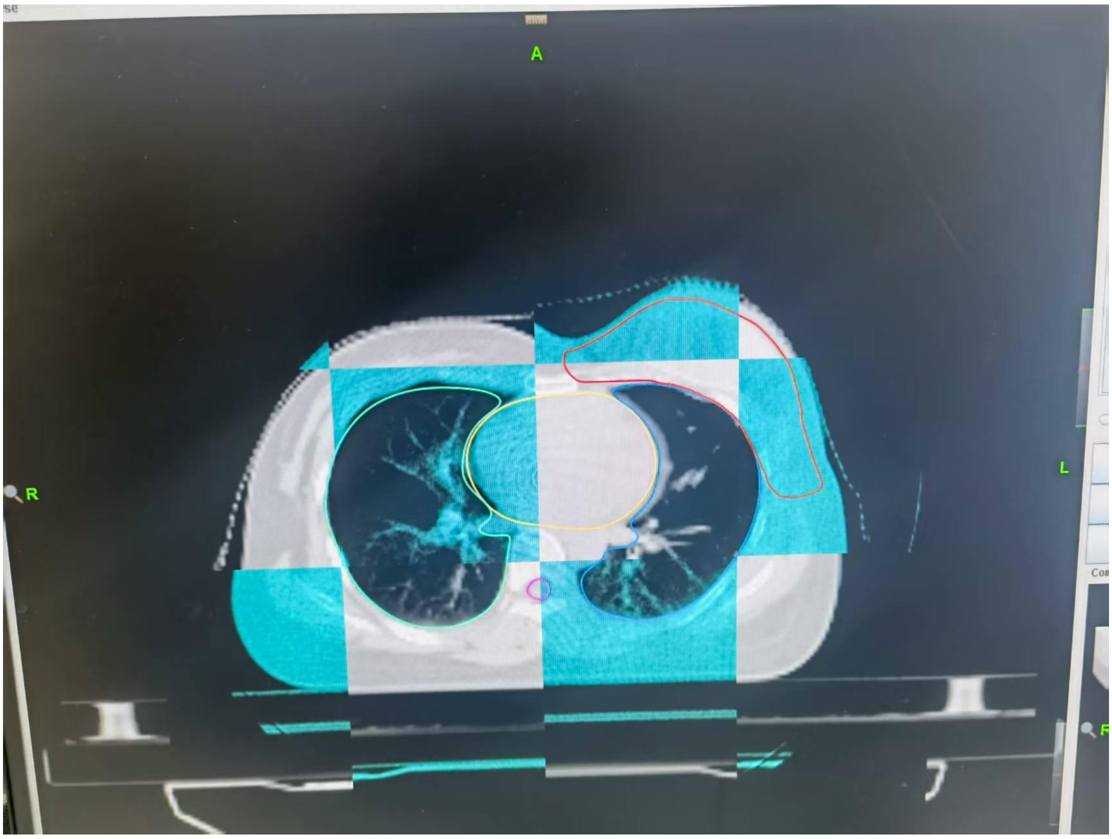

Dose prescription

A tissue-equivalent bolus of 5 mm was used to irradiate the chest wall of the patients who underwent a radical mastectomy. The bolus is intended to optimize the coverage of the chest wall by the 95% isodose, thereby increasing the surface radiation dose. The total dose of 50 Gy was prescribed for each PTV over 25 fractions (2 Gy per fraction). All treatment plans were computed utilizing the TomoHelical mode, which serves as the original beam delivery technique integrated into the TOMO System and enables rotational delivery of a fan beam. The plans were evaluated with reference to the ICRU criteria of 95% of the target volume getting covered with 95% of the prescribed dose with minimum spillage to the surrounding normal tissue. In order to achieve this, the dose prescription was modified in most patients. Dose prescriptions in most patients were such that 95% of the PTV will receive 47.5 Gy dose, i.e. 95% of the prescribed dose 50 Gy.

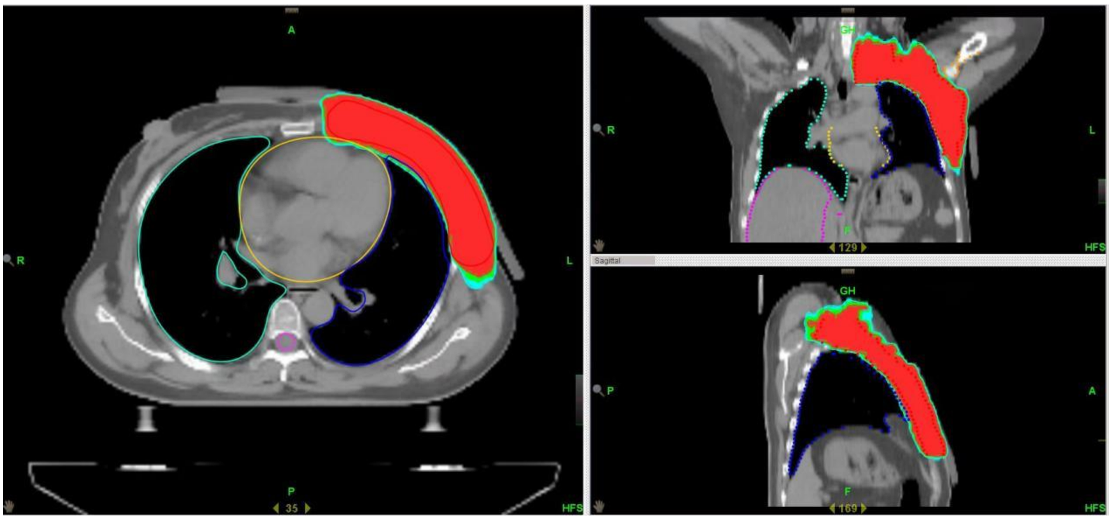

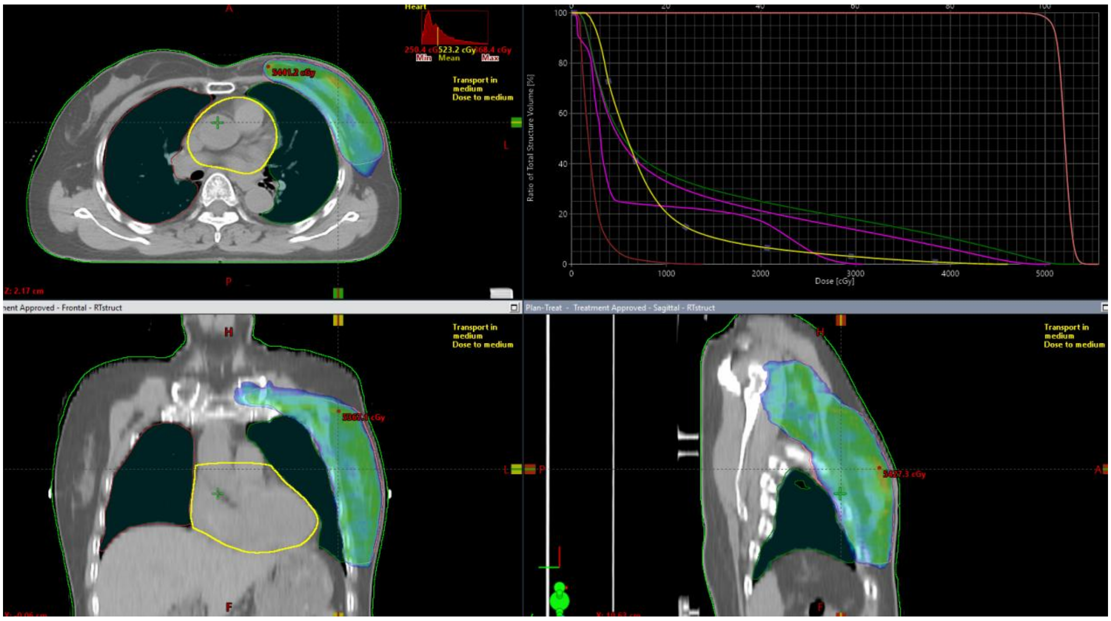

Treatment plan evaluation

Treatment plans were calculated with the Tomotherapy planning system. When setting OAR doses, we considered international recommendations (QUANTEC—Quantitative Analysis of Normal Tissue Effects in the Clinic, 2010). For the lung, the dose limits were V5Gy < 60%, V20Gy < 30%, and V25Gy < 10%. For the heart, the dose limit was V40Gy < 5%. We also took into account the PTV coverage conformity, homogeneity indices, and the values of mean, minimum and maximum doses with V90%, V95%, V107%, and V110% of the prescribed dose.

The conformity index (CI) was measured by:

$$CI=TV/PTV$$

PTV, planning target volume; TV, treated volume.

The homogeneity index was measured by following formula

$$HI=(D2\%-D98\%)/D50\%$$

A lower HI value serves as an indicator of a more homogeneous distribution across the PTV. The near minimum D98% and near maximum doses D2% are serves as doses received by 98% and 2% of the PTV volume, respectively.

IMRT planning

The IMRT treatment planning was performed with the Eclipse, version 16.1 (Varian medical Systems Inc, Palo Alto, USA) . Patients treated with conventionally IMRT received a total dose of 50 Gy in 25 fractions, followed by an RT boost on the surgical bed of 10 Gy in 5 fractions. Dose was delivered with wedged photon tangential fields, and boost was treated with an electron direct field. The OARs constraints were that 5% of the heart and 20% of the lung were kept to < 20 Gy. Homogeneity of the dose to the target was controlled by keeping the maximum dose within 55 Gy and the volume receiving more than 52.5 Gy (V52.5) < 10%.

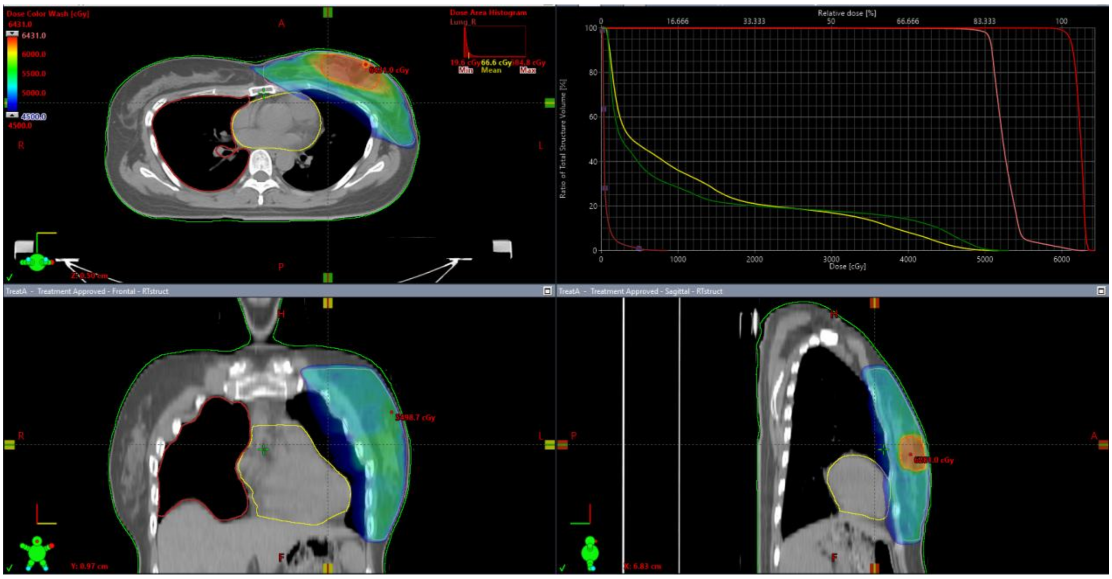

A dose of 50 Gy in 25 consecutive once-daily fractions was prescribed. The following constraints were adopted for plan optimization: PTV coverage, 100% of PTV covered by 95% of the prescribed dose (V47.5 = 100%); maximal dose to PTV < 107% (53.5 Gy); minimal dose to PTV 45 Gy; uninvolved breast: not > 50% received a dose of > 50% of the prescribed dose (V25 < 50%); ipsilateral lung, not > 20% received a dose > 10 Gy (V10 < 20%); contralateral lung, not > 10% received a dose > 5 Gy (V5 < 10%); contralateral breast, maximal dose < 1 Gy; and heart, not > 10% received a dose > 5Gy (V5 < 10%).

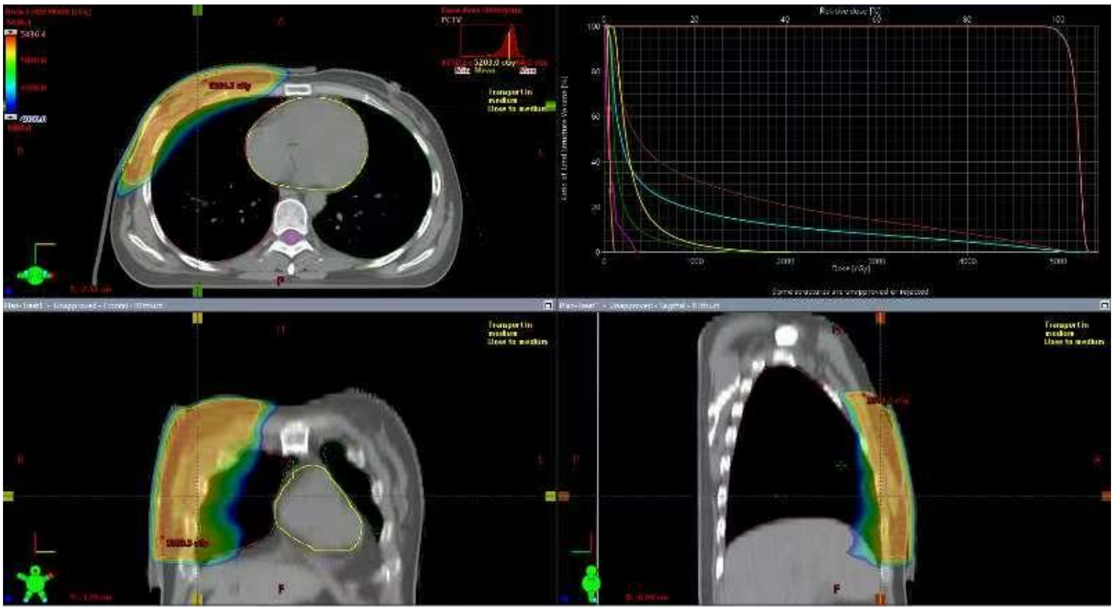
Relative dose [%]
Dose [cGy]
Some structures are unapproved or rejected
Transport in medium
Dose to medium
5203.0 cGy
